# Delphi Consensus on the Management of Spanish Patients with Post-Stroke Hemiplegic Shoulder Pain Treated with Botulinum Toxin A: Result Study

**DOI:** 10.3390/toxins17010040

**Published:** 2025-01-16

**Authors:** Carlos Cordero-García, Irene de Torres, Jacobo Formigo-Couceiro, Lluis Guirao, Mª Dolores Romero-Torres, Sergio Otero-Villaverde, Alberto Herrera, Cristina Santa, Antonio Mena-Rodriguez

**Affiliations:** 1 Juan Ramón Jiménez University Hospital, Ronda Exterior Norte s/n, 21005 Huelva, Spain; ccordero.rhb@gmail.com; 2 Reina Sofía University Hospital, Avda. Menéndez Pidal s/n, 14004 Córdoba, Spain; detorres.irene@gmail.com; 3 University Hospital of A Coruña, As Xubias, 84, 15006 A Coruña, Spain; jacobofor@gmail.com (J.F.-C.); sergiooterov@hotmail.com (S.O.-V.); 4 Mútua Terrassa University Hospital, Plaça del Doctor Robert, 5, 08221 Terrassa, Spain; lguirao@mutuaterrassa.cat; 5 Virgen Macarena University Hospital, Avda. Dr. Fedriani, 3, 41009 Sevilla, Spain; lolart10@hotmail.com; 6 Ipsen, Avda. Burgos, 21, 28036 Madrid, Spain; alberto.herrera@ipsen.com (A.H.); cristina.santa@ipsen.com (C.S.); 7 Doctor Negrín University Hospital of Gran Canaria, Pl. Barranco de la Ballena s/n, 35010 Las Palmas de Gran Canaria, Spain

**Keywords:** Delphi method, hemiplegic shoulder pain (HSP), post-stroke, spasticity, botulinum toxin A, abobotulinumtoxinA

## Abstract

The study aimed to identify expert opinions and obtain recommendations on the management of post-stroke hemiplegic shoulder pain (HSP) and treatment with botulinum toxin A (BoNT-A). A multicenter Delphi study was conducted using an online survey designed by a committee of experts with at least 10 years of experience in post-stroke HSP management with BoNT-A in Spain. Forty-seven panelists (specialists with at least 5 years of experience in post-stroke HSP management with BoNT-A) rated their level of agreement in two rounds based on acceptance by ≥66.7% of them. In round 1, 245 statements on three dimensions were evaluated (diagnosis, treatment, and follow-up of the HSP patients treated with BoNT-A). A total of 159 statements (70.9%) were finally accepted after round 2. Experts recommended BoNT-A as soon as spasticity affects daily activities. They considered ultrasound as the preferred guided technique. Experts recommended regular assessments using validated scales and patient-reported outcomes to evaluate treatment goals and safety. In case of lack of response, experts suggested increasing the dose or number of treated muscles or considering alternative treatments. These consensus-based recommendations offer clinicians an approach to the management of post-stroke HSP with BoNT-A, supporting informed decision making.

## 1. Introduction

### 1.1. Plain Language Summary

This study gathered opinions from experts on how to manage shoulder pain due to spasticity (disruption in muscle movement patterns) in patients who have experienced a stroke. The experts focused on using a treatment called botulinum toxin A (BoNT-A).

A group of specialists with at least 5 years of experience in treating post-stroke shoulder pain with BoNT-A took part in an online survey, following a methodology called the Delphi consensus. They shared their views on diagnosing, prognosing, treating, and following-up on patients.

As a recommendation, the experts suggested initiating treatment as soon as spasticity begins to affect daily activities and prefer using ultrasound-guided injection techniques for BoNT-A administration to improve prognosis and outcomes. Regular follow-ups were advised, employing standard assessment scales and patient feedback to evaluate treatment efficacy and safety. In cases where the treatment proves inadequate, experts proposed several strategies: increasing the BoNT-A dosage, expanding the treatment to include additional muscles, or exploring alternative therapeutic options.

These recommendations can help doctors make informed decisions when treating shoulder pain in stroke patients using BoNT-A. This may lead to better care for people facing this challenging condition.

### 1.2. Introduction

Hemiplegic shoulder pain (HSP) is one of the most common medical complications following stroke [1]. Data from various studies indicate that between 30–65% of all stroke patients develop shoulder pain, with a prevalence of up to 39% at one year [2,3,4,5]. HSP affects rehabilitation and impacts the patient’s quality of life (QoL) [2]. In many cases, patients may develop chronic HSP due to structural injuries and postural impairment [6].

Spasticity can develop in the context of various neurological conditions, including the post-stroke process [7]. It can manifest at any time but is typically observed between 1–6 weeks following a stroke [8]. Spasticity has been defined as an alteration in sensor-motor regulation following injury to the upper motor neuron, resulting in involuntary intermittent or continuous muscle activity [9]. The upper limbs are affected more frequently than the lower limbs (18% vs. 5.5%) [10]. Risk factors for the development of spasticity include early and severe paresis, pain, and sensory deficits [10]. Spasticity significantly contributes to pain in the hemiplegic shoulder through mechanisms including postural alteration, glenohumeral subluxation, and contracture formation [1]. As a result, spasticity and associated pain have a significant impact on a patient’s daily life [11]. Spastic HSP typically appears in a position of adduction and internal rotation. Spasticity, loss of muscle strength, and motor control alter the movement of the shoulder joint [12]. Botulinum toxin A (BoNT-A) has been established as a well-tolerated treatment for spasticity, as it reduces focal spasticity, improves functionality, and decreases pain [12]. Specifically in focal spasticity of the upper limb, BoNT-A is considered the first-line treatment choice in clinical practice, as it offers the opportunity to target specific muscles [13]. It provides the possibility of a single, focal, reversible treatment tailored to the patient. It is not a standalone therapy, as it is accompanied by complementary physical therapies such as active or passive physiotherapy techniques, which enhance the treatment outcomes [14]. More specifically, treatment with abobotulinumtoxinA (aboBoNT-A, Dysport^®^) has been investigated for its potential to alleviate HSP and improve upper-limb function in adults with post-stroke spasticity [15]. A phase 3, open-label study evaluated the efficacy of aboBoNT-A in this patient profile. The results demonstrated significant improvement in pain as measured by a decrease of 0.7 points on the Disability Assessment Scale (DAS), and enhanced active function, with an increase of 0.60 points on the Modified Frenchay Scale (MFS) [16].

However, in clinical practice, the use of BoNT-A in spastic HSP is highly variable. A survey of clinical practice among healthcare professionals specializing in the treatment of spasticity showed that 86.8% would consider injecting BoNT-A for the treatment of HSP associated with spasticity, but only 8.8% would choose BoNT-A as first-line treatment, and 54.4% agreed or strongly agreed that BoNT-A is an effective treatment [17].

To provide appropriate BoNT-A treatment for HSP related to spasticity, a comprehensive evaluation is necessary to determine the involved muscles. Knowing the dominant spasticity pattern in the shoulder helps define the muscles to be treated [12]. In their study, Hefter et al. found that four out of the five most common spasticity patterns in the upper extremity involve adduction and internal rotation of the shoulder. According to published data, the following muscles can be identified as causing adduction and internal rotation: pectoralis major, latissimus dorsi, teres major, and subscapularis [18,19,20]. However, it is unclear which of the identified muscles are the most important to inject, and the injection parameters used in clinical practice are heterogeneous [20,21]. Clinical experience and muscle accessibility are usually the dominant factors that determine the strategy of BoNT-A treatment in spastic HSP after stroke [21].

Therefore, the management of spastic HSP after stroke retains several controversies and ongoing debates, namely treatment with BoNT-A in this context. This highlights the need for a consensus study involving experts on these topics. The RESULT (Recommendations on Post-Stroke ShoULder Treatment) project was a multicenter study conducted in Spain using the Delphi method in two rounds to identify expert opinions and obtain recommendations on the management of post-stroke spastic HSP and treatment with BoNT-A.

## 2. Results

### 2.1. Expert Panel

A total of 47 specialists distributed throughout Spain participated as Delphi panelists in rounds 1 and 2 (Figure S1). The Delphi expert panel was selected by the scientific committee based on criteria including specialization in Rehabilitation and Physical Medicine, significant clinical experience with neurological patients, at least 5 years of experience in treating hemiplegic painful shoulder and using botulinum toxin under ultrasound guidance, and membership in the Spanish Society of Rehabilitation and Physical Medicine (SERMEF). The characteristics of the panelists are summarized in Table 1. Briefly, the median (range) of professional experience of the participants was 22 years (4.0–41.0). The median (range) of the number of neurologically affected patients per week visited by the panelists was 20 (6.0–75.0). Finally, most of the participants were available for a specific spasticity consultation (n = 36, 76.6%).

### 2.2. Results from Delphi Rounds 1 and 2

**Figure 1:**
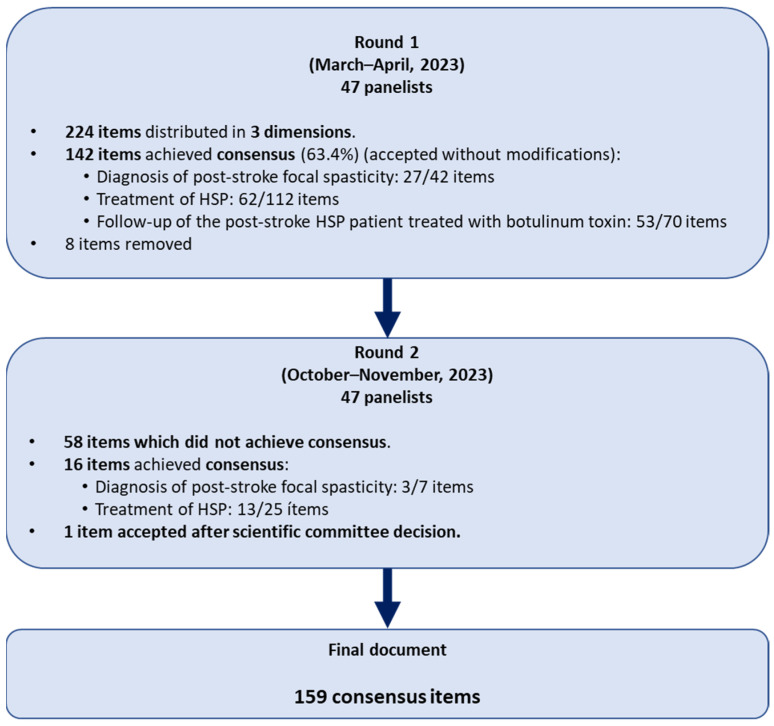
Results of the Delphi study.

Figure 1 illustrates the results of the Delphi study. In round 1, panel members evaluated 224 items from the dimensions of diagnosis (dimension 1), treatment (dimension 2), and follow-up (dimension 3) of the HSP patients treated with BoNT-A.

Consensus was reached for 142 items (63.4%) in round 1, which were accepted without modifications. After the scientific committee meeting, eight items were removed. The items that did not meet consensus or were reformulated after round 1 were put forward for inclusion in round 2. Sixteen items reached an agreement in round 2. Fifty-eight items did not achieve consensus in round 2, and after discussion, the scientific committee decided to accept one item. At the end of the Delphi process, a total of 159 items (70.9%) were finally retained. Consensus was reached on 31 (73.8%), 75 (66.9%), and 53 (75.7%) of the statements for each dimension.

**Figure 2:**
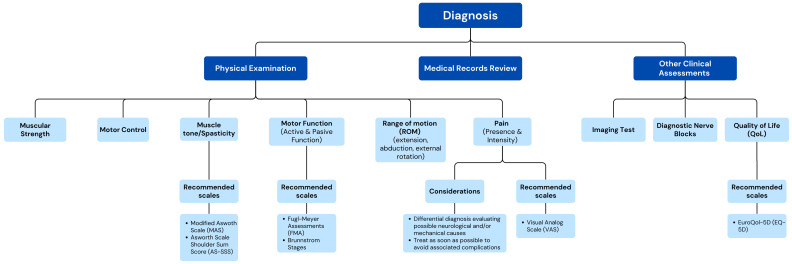
Conceptual map based on the results of the two-step Delphi process for the items regarding to the diagnosis of post-stroke focal spasticity (Dimension 1).

**Figure 3:**
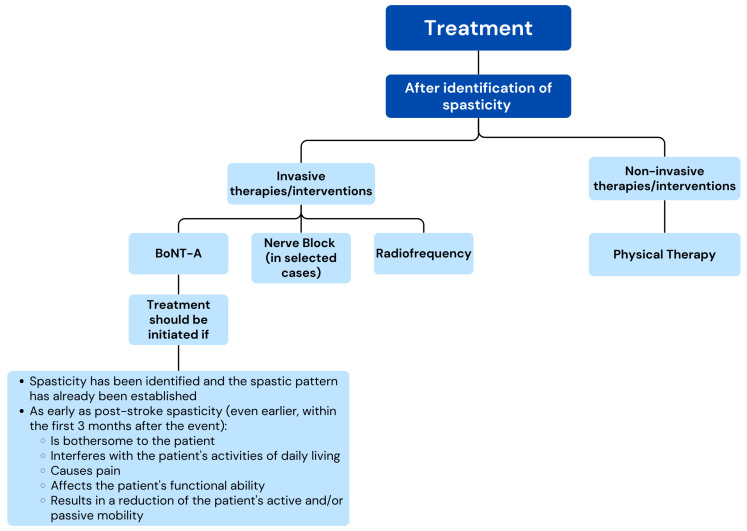
Conceptual map based on the results of the two-step Delphi process for the items regarding the treatment of post-stroke HSP (Dimension 2).

**Figure 4:**
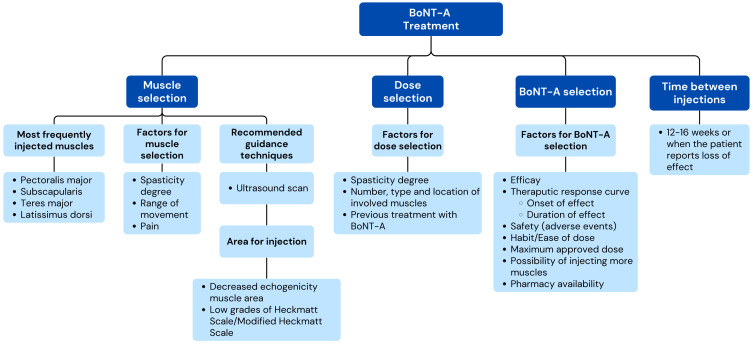
Conceptual map based on the results of the two-step Delphi process for the items regarding the treatment of post-stroke HSP with BoNT-A (Dimensions 2 and 3).

Supplementary Tables S1–S3 summarize the results from the Delphi process and the level of agreement after the two rounds for the statements related to each dimension. To simplify the interpretation of the results, sets of recommendations were developed, in the form of conceptual maps, with the most relevant items that reached expert consensus in the Delphi process (Figure 2, Figure 3 and Figure 4).

#### 2.2.1. Dimension 1: Diagnosis of Post-Stroke HSP

Consensus was reached for 31 items (73.8%) (Table 2 and Table S1, Figure 2).

The results concluded with a consensus of 76.6% that there are no well-established criteria for the diagnosis of post-stroke HSP. However, there was agreement among the experts that the diagnosis should primarily include a review of the medical history (100%), physical examination (100%), ultrasound (76.6%), radiography (68.1%), and diagnostic blockades (65.2%). Additionally, the initial assessment of a patient with post-stroke HSP should include the evaluation of spasticity (100%), pain (100%), muscle balance (97.9%), motor control (97.9%), range of motion (ROM) (97.9%), active/passive functionality (95.7%), and QoL (89.4%). Specifically, regarding pain, there was 100% consensus that it is important to treat post-stroke HSP as early as possible to avoid major complications. The scales that reached consensus among the experts for the different assessments were the visual analog scale (VAS) (93.6%) for pain; the Modified Ashworth Scale (MAS) (100%) and the Ashworth Scale Shoulder Sum Score (AS-SSS) (70.2%) for muscle tone or spasticity; the Fugl–Meyer Assessment (FMA) (68.1%); the Brunnstrom stages (74.5%) for motor function recovery; and the EQ-5D (70.2%) for quality of life.

#### 2.2.2. Dimension 2: Treatment of Post-Stroke HSP

Consensus was reached for 75 items (66.9%) (Table 3 and Table S2, Figure 3).

After the identification of spasticity in post-stroke HSP, the experts reached a consensus that non-invasive interventions would primarily include physical therapy (89.4%). Among the invasive interventions, there was agreement to evaluate injection with BoNT-A (100%), nerve block (89.4%), and radiofrequency (78.7%), while surgical intervention was discouraged (95.7% disagreement).

##### BoNT-A Treatment

Regarding BoNT-A treatment, the experts reached broad consensus in recommending starting treatment as soon as the disorder causes interference with daily activities (100%), causes pain (97.9%), or results in a reduction of active/passive mobility (93.6%), even within the first 3 months after stroke (Figure 4). The muscles most frequently considered for injection are the pectoralis major (100%), subscapularis (95.7%), latissimus dorsi (68.1%), and teres major (68.1%) (Table 4). The experts disagreed that difficulty in accessing the muscle is a factor to be considered for selecting the muscle to be injected (76.1% consensus in disagreement). In this regard, the panelists were close to agreeing (without reaching consensus) that the subscapularis (55.6%) is the muscle that presents the greatest difficulty in access for injection. Participants recommended that, in most cases, the administration route for BoNT-A should be intramuscular (97.9%), with at least two points of injection in the case of the pectoralis major (74.5%) and latissimus dorsi (74.5%) and with a single point of injection in the case of the subscapularis (95.7%) and teres major (91.5%).

As guided techniques to improve the precision of BoNT-A administration, the experts considered the use of ultrasound (100%), while electromyography (82.2% disagreement) and electrical stimulation (78.3% disagreement) were discouraged. Ultrasound muscle echogenicity to guide infiltration was recommended (76.1%), considering the areas of lower echogenicity (85.1%) or using the Heckmatt scale (83.0%) or the modified Heckmatt scale (83.0%).

Regarding the BoNT-A dosage to manage post-stroke HSP, the experts recommended considering the spasticity degree (95.7%); the number, type, and location of the muscles involved (95.7%); and previous BoNT-A treatment (76.6%). In contrast, the echogenicity degree (78.3% disagreement) and the physician’s experience (67.4%) were not regarded as factors to be considered. The experts agreed that the duration of the therapeutic response (91.3%), the safety profile (91.3%), and maximum allowed dose (91.3%) are the most important factors in choosing a specific BoNT-A type.

Regarding aboBoNT-A administration, the panelists agreed that, in general, >1 mL should not be administered at a single injection point (67.4%) (Table 5). The dilution for each 500 U of aboBoNT-A should be 2 mL (82.6%). The dose range for the specific treatment of HSP with aboBoNT-A should be 600–1200 U (67.4%), with 1200–1500 U being discouraged (69.6% disagreement), although the experts agreed that, in certain cases, higher doses per muscle than indicated in the summary of product characteristics (SmPC) could be recommended (80.4%). The recommended dose for injection per session in each muscle should be, according to the experts, 150–300 U in all cases (pectoralis major, subscapular, and latissimus dorsi), with an optimal time between infiltrations of 12–16 weeks (80.0%). In this sense, a new session could be evaluated from 12 weeks onwards, when the patient reports that the effect has decreased or disappeared (73.9%). BoNT-A injection should be accompanied by home exercises (91.3%) or physiotherapy (80.4%), and combining it with orthoses is discouraged (73.9%).

#### 2.2.3. Dimension 3: Follow-Up of Post-Stroke HSP

**Figure 5:**
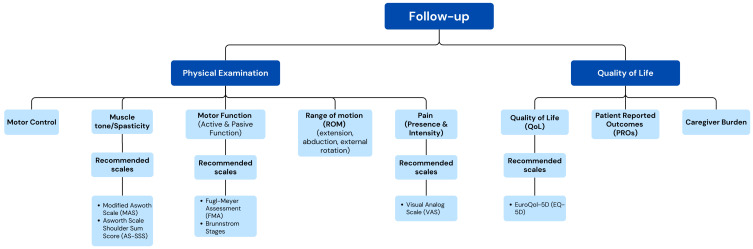
Conceptual map based on the results of the two-step Delphi process for the items regarding follow-up of post-stroke HSP management (Dimension 3).

**Figure 6:**
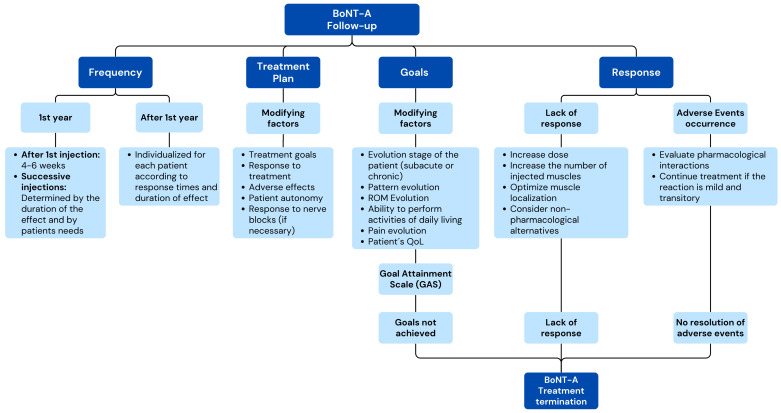
Conceptual map based on the results of the two-step Delphi process for the items regarding follow-up of post-stroke HSP with BoNT-A (Dimension 3).

Consensus was reached for 53 items (75.7%) (Table S3, Figure 5 and Figure 6).

Overall, 93.5% and 78.3% of the experts agreed that it is recommended to use validated scales and patient-reported outcomes (PROs), respectively, to assess patient evolution, and caregiver burden, if applicable (93.5%) (Figure 5). As in the initial patient stage, the panelists agreed to assess pain by using the VAS (97.8%), MAS (100%), and AS-SSS (76.1%) for muscle tone or spasticity assessment and the EQ-5D (73.9%) for QoL assessment.

##### BoNT-A Treatment Follow-Up

The participants agreed that the first follow-up after the first BoNT-A injection should take place 4–6 weeks after the procedure (95.7%) and that subsequent follow-ups should be performed every 12–16 weeks during the first year (84.8%), and then, they should be individualized for each patient (97.8%) (Figure 6). Independently of these considerations, the frequency should be determined by the duration of the treatment response (73.9%) and the patient’s needs (73.9%). During follow-up visits, the treatment goals should be re-evaluated based on the Goal Attainment Scaling (GAS) (84.8%) and considering, among other aspects, goals, treatment response, and safety (97.8%). In the case of the evolution of patients treated with BoNT-A, consensus was also reached to use the Modified Tardieu Scale (71.7%) for spasticity evolution and the Brunnstrom stages (71.7%) for motor function recovery assessment.

All the experts agreed that before the next BoNT-A injection, it is necessary to inquire about the incidence of possible side effects of the previous administration, and in case there were any effects, 93.5% of the participants recommended evaluating possible drug interactions, and 76.1% recommended continuing treatment if the side effects are mild and transient.

In case of possible lack of response, the panelists agreed to increase the dose (89.1%), increase the number of treated muscles (84.8%), optimize the muscle location to inject (95.7%), increase both the dose and the number of injected muscles (87%), or consider a non-pharmacological treatment alternative (69.6%).

## 3. Discussion

Stroke is a common condition worldwide and considered to be the third leading cause of disability [22]. HSP is the most common pain condition in post-stroke patients and one of the four most common medical complications after a stroke, along with depression, falls, and urinary tract infections [1,23]. Consequently, the appropriate management of HSP is of great clinical relevance. However, the identification, diagnosis, and management of post-stroke HSP remain a challenge for specialists. In this context, the Delphi method is important in medical science, as it allows for the collection of expert opinions, promotes reflection and the exchange of ideas, reaches a consensus, and ensures the reliability of results [24,25,26].

The most significant predictors of HSP following stroke include a younger patient age (≤70 years), female gender, a history of relevant medical conditions, hemorrhagic stroke, left-sided hemiparesis, hemi-spatial neglect, a high level of disability (National Institutes of Health Stroke Scale score > 14/42), sensory impairment, and spasticity [3]. This is in line with the opinion of the panelists in this study, in which the risk factors with the highest level of agreement were the impairment of voluntary motor control, spasticity, proprioceptive dysfunction, the range of motor restrictions, tactile extinction, and a history of prior anomalies. Although the present study did not reach consensus on age, the latter was the strongest non-modifiable risk factor for ischemic stroke [3]. Gender, on the other hand, was not included as a statement in this case, but the differences are evident in relation to pain sensitivity, functional status, and comorbidities. Thus, female gender is a significant risk factor for post-stroke pain [3,27].

Regarding the diagnosis of post-stroke focal spasticity, the results of this Delphi study are consistent with the information found in the literature. In this regard, the VAS or the Numeric Rating Scale (NRS) are primarily reported as a widely used instruments to easily assess pain in the spastic shoulder [12]. Regarding evaluation of spasticity, the MAS is generally the most widely used scale at function level, which aligns with the consensus opinion expressed by the expert panel participating in this Delphi study. However, there are some doubts about whether this scale, which is widely used in clinical trials, indirectly measures spasticity [12]. Other characteristics, such as hypertonicity, muscle spasms, or spastic dystonia, among others, are not measured by the MAS [28]. Independent evaluations of other scales, such as the Modified Tardieu Scale (which did not reach consensus on its use in the present study) or the Fugl–Meyer Assessment, among others, also suggested a lack of reproducibility [29]. Therefore, it is necessary to evaluate new and appropriately sensitive scales or to consider different scales to assess the various aspects involved. Notably, the Spasticity-related Quality of Life 6-Dimensions (SQoL-6D) scale, which did not achieve consensus, is worth mentioning. Specifically designed to evaluate the impact of upper-limb spasticity due to any cause on a patient’s health-related quality of life, the SQoL-6D has been validated to accurately capture the burden of spasticity in patients with this condition [30,31].

Other techniques, such as ultrasound of spastic muscles, provide relevant information about non-neuronal factors that contribute to spasticity [32]. In the present study, as guided techniques to improve the precision of BoNT-A administration, the experts considered the use of ultrasound (100%). Ultrasound provides macroscopic information about changes in the morphological properties of muscles related to post-stroke hemiparetic spasticity [33]. The panelists reached consensus that ultrasound muscle echogenicity to guide infiltration is recommended considering the areas of lower echogenicity by using the Heckmatt scale or the modified Heckmatt scale. This could be considered controversial since, in principle, the areas of greater spasticity would be those with greater fibrosis and with more echogenicity zones, but it agrees with published data [34]. Therefore, despite the existence of some potentially promising techniques, the evaluation of spasticity and the identification of spasticity-related muscles remain a challenge.

Several interventional and non-interventional approaches have shown promise in post-stroke HSP management. Nerve blocks, specifically suprascapular, have demonstrated efficacy in reducing pain and improving ROM [35]. Radiofrequency treatments have also emerged as potential options for pain relief and functional improvement [36]. Physical therapy remains a cornerstone of treatment, often combined with other modalities to enhance the outcomes [37,38]. More recently, cryo-neurolysis has gained attention as a minimally invasive technique for managing chronic pain conditions, though its specific application in HSP requires further investigation [39,40]. The integration of these approaches, tailored to the individual patient needs, may offer a comprehensive strategy for improving overall rehabilitation outcomes.

Regarding BoNT-A treatment, the results presented here indicate that the muscles most frequently considered for injection are the pectoralis major, subscapularis, and latissimus dorsi, and the panelists disagreed that difficulty in accessing the muscle should be a factor for selecting the muscle to inject. Previous research has involved injecting BoNT-A into the adductor muscles of the shoulder (pectoralis major, subscapularis, teres major, and latissimus dorsi), and the resulting outcomes support the muscular etiology of the pain, which is thought to arise from joint movement disorganization mediated by an imbalance between adductor and abductor muscles [15,41]. A post hoc analysis in patients with chronic post-stroke spasticity revealed that, using the Goal Attainment Scaling (GAS), most of patients (72.1%; 95% confidence interval: 57.2–83.4%) achieved their goal of reducing shoulder pain after a single treatment cycle of aboBoNT-A [15]. Moreover, the experts considered that the administration route should be intramuscular in most cases, and they recommended the use of ultrasound as guiding techniques to improve the precision of BoNT-A administration.

The results concerning muscle selection are in line with those previously published. In this sense, the pectoralis major was identified as the most targeted muscle for injection in patients with adduction and internal rotation as shoulder spastic patterns [42]. Other commonly selected muscles, according to the literature, are the subscapularis, latissimus dorsi, and teres major [19,42,43,44]. The literature suggests that ultrasound-guided injection of the subscapularis muscle is a safe, precise, and effective technique, although it presents a greater degree of difficulty [45]. However, the order of preference for the different muscle groups differs among studies, indicating differences in routine clinical practice and suggesting that homogeneous indications would be advisable to ensure greater effectiveness.

Regarding the use of guidance methods in shoulder muscle injection, the diversity of results published by different works reflects important differences in the use of methods in actual clinical practice. Use of the ultrasound scan is relatively recent, although it has been growing rapidly [42,46]. The unanimity of recommendation in our results contrasts with the use of other methods, such as anatomical landmarks, electrical stimulation, or electromyography in routine practice in countries such as Portugal or Canada, which were discouraged here by the participating panelists [42,43].

The participants agreed that the dose range for the specific treatment of HSP with aboBoNT-A should be 600–1200 U, with a recommended dose per session in each muscle of 150–300 U, spacing the infiltrations every 12–16 weeks (depending on whether the patient reports a decrease or disappearance of the effect). This agreement aligns with the post hoc analysis of a real-world evidence study that found a median dose of 600 U, with a maximum of 1200 U in cycle 1, that increased to 1500 U in cycle 2 [15]. Recent experience in clinical practice in Portugal reflects the use of different maximum doses depending on the selected muscle, with generally lower values [42]. However, this is expected since individualized dosages of BoNT-A are recommended for each patient depending on spasticity severity, therapy goals, and other considerations, resulting in anticipated variability [47].

Appropriate patient follow-up is crucial for the management of post-stroke HSP and for the optimization of the treatment. In this sense, the results presented here showed that, while it may be important to set a frequency of visits, especially in the first year, this should be determined by the individual needs of the patient. According to our results, follow-up should be based on three aspects: (1) goal scales to assess aspects such as pain and spasticity (using VAS, MAS, and AS-SSS); (2) PROs to assess QoL (EQ-5D) and caregiver burden; and (3) treatment outcomes assessment, using the Modified Tardieu Scale (spasticity), the Brunnstrom stages (motor function), and GAS (treatment goals).

The use of scales such as VAS, MAS, and AS-SSS and their reliability are discussed above. The GAS is a recognized tool for assessing the success of pre-established treatment goals [48]. It is receiving increased interest in clinical practice since it allows for the assessment of therapeutic efficacy based on goals established by the patients themselves rather than using generic scales, which may not always contain the condition that is most relevant to the patient [48,49]. The GAS may be used to address all aspects of the International Classification of Functioning Disability and Health, including activity, participation, quality of life, and environmental considerations [48].

Finally, the results reflected two key points in the follow-up of post-stroke HSP patients treated with BoNT-A: the assessment of possible treatment-related adverse effects and a lack of response. In this regard, the panelists concluded that, when adverse effects occur, a possible drug interaction should be assessed, and the majority agreed to continue the treatment if the adverse effects are mild or transient. In the absence of response, the participants agreed to optimize muscle localization, increase the dose and/or the number of muscles infiltrated, and consider a non-pharmacological alternative in this order in terms of percentage of agreement.

Despite the potential risks associated with the injection and the toxin itself, experience with BoNT-A indicates that adverse effects are rare and, in most cases, mild [42]. BoNT-related adverse effects such as injection site discomfort, bruising, and muscular weakening are often mild, while significant systemic adverse effects are rare [50,51].

Regarding the lack of response to BoNT-A, the results agreed by the participants in the present study were in line with the current trend in the follow-up management of patient response to BoNT-A treatment. Based on this, the common actions if the treatment presents a lack of response include reassessing the patient’s spasticity and muscle involvement, optimizing BoNT-A administration (by refining the timing of injections, the targeted muscles, and the dosing), addressing adherence to BoNT-A therapy over time, and considering alternative treatment options (oral medication, physical/occupational therapy, kinesio taping, shock wave therapy, anesthetic diagnostic nerve block, thermal radiofrequency, and surgical options for refractory cases) [12,50,52,53,54,55,56]. Similarly, other studies relying on expert consensus have suggested that in cases of suboptimal efficacy (without safety concerns), the dose, target muscles, number of muscles treated, number of injection sites, the injection technique, and/or dilution should be reevaluated at the next treatment visit in order to optimize outcomes [33,57]. One of the main strengths of our study is that it included a large and geographically dispersed group of physicians, ensuring a representative sample of the specialists that use BoNT-A to treat post-stroke HSP. In addition, this work addresses post-stroke HSP diagnosis, treatment, and follow-up, which are three key points in the management of this patient profile. Notably, this Delphi study culminates in the development of a practical conceptual map, which translates the expert consensus into a clear, actionable clinical practice guideline for the effective management of post-stroke HSP in real-world clinical settings.

The limitations of the present study are related to the Delphi methodology itself. The fact that experts express a high degree of agreement does not directly imply that a recommendation is necessarily effective or possible to develop in routine clinical practice. On the other hand, response bias is possible, and the participants could have expressed their agreement or disagreement based on the possibilities offered by their experience or usual clinical practice and not on what would be uniformly advisable. In this sense, the results of the present study represent the starting point for the development of recommendation documents and management guidelines.

## 4. Conclusions

In conclusion, the consensus among experts underscores the importance of initiating BoNT-A treatment promptly when spasticity begins to interfere with daily activities, causes pain, or diminishes active or passive mobility, even within the first three months post stroke. Ultrasound guidance is recommended as the preferred technique for administering BoNT-A. Regular assessments utilizing validated scales and PROs are essential for monitoring treatment efficacy and safety. In cases of inadequate response, the experts advocated for dose escalation, increasing the number of treated muscles, or exploring alternative therapeutic options. These recommendations aim to optimize patient outcomes and enhance quality of life for individuals experiencing post-stroke spasticity. The expert consensus recommendations derived from this Delphi study may provide support and guidance on clinical decision making regarding the diagnosis and follow-up of post-stroke HSP and for carefully weighing the potential benefits of treatment with BoNT-A.

## 5. Materials and Methods

### 5.1. Study Design

The study consisted of an online survey completed by Spanish physicians specialists in physical medicine and rehabilitation and was endorsed by the Spanish Society of Rehabilitation and Physical Medicine (SERMEF). Approval by the Institutional Review Board (IRB) or equivalent ethics committee(s) was not required, as this Delphi study did not involve human subject research. No patient data were collected for this study, which was based entirely on the feedback and opinions provided by experts.

The Delphi process is a widely accepted scientific method of systematic information collection from a group of experts (termed the Delphi expert panel) on controversial or complex topics [58]. Each panel expert provides opinions individually and anonymously without the biasing effect of dominant individuals or group pressure [24,59,60]. The Delphi process ends when an agreement has been reached on the discussed topics.

### 5.2. Delphi Process

#### 5.2.1. Selection of the Delphi Participants

The expert scientific committee comprised seven specialists experienced in managing patients with post-stroke HSP and recognized experts in the field (at least ten years of experience in post-stroke HSP management with BoNT-A).

A total of 47 specialists from hospitals distributed across Spain were invited to participate in the project as members of the Delphi expert panel (panelists) in both round 1 and round 2 of the Delphi process (Supplementary Figure S1). The panelists were selected by the scientific committee based on their extensive experience and knowledge of the management of patients with post-stroke HSP. No offers of incentives were made to participants.

The panelists were provided by e-mail with an informative leaflet outlining the aims and the study procedure and including an electronic link to the online survey. The purpose of the panel of experts was to reach a consensus based on the current clinical evidence and their daily practice in and knowledge of the management of patients with post-stroke HSP.

#### 5.2.2. Selection of the Delphi Questionnaire Dimensions and Items

The scientific committee carried out a systematic literature review regarding the main topics, focusing on current controversial and unmet issues. After a careful and critical review of the selected literature and based on their knowledge of the clinical management of the pathology, the scientific committee developed the first set of domains and statements or items for the Delphi questionnaire.

#### 5.2.3. Round 1

Between March and April 2023, the panelists were asked to rate their level of agreement with each questionnaire item on a 9-point Likert scale from 1 (completely disagree) to 9 (completely agree). Each item was categorized according to the scores as rejected (scores 1–3), undetermined (scores 4–6), or accepted (scores 7–9). The panelists were also encouraged to provide comments after scoring each item using open-text comment fields included in the online survey.

After analyzing the data obtained from the first Delphi round, the scientific committee experts participated in a teleconference meeting, where the Delphi survey results were presented and discussed. Item selection was based on the acceptance of questionnaire items by ≥66.6% of the expert panel and the agreement of the scientific committee. Statements not achieving 66.6% agreement were removed or modified according to the feedback provided by the expert panel. All statements were assessed given the experts’ suggestions. After round 1 was completed and the expert comments summarized, amendments were made to some questionnaire items. The updated questionnaire was redistributed to the panelists for round 2.

#### 5.2.4. Round 2

In round 2, between October and November 2023, the same panelists were asked to evaluate the list of items that did not meet consensus from round 1, using the same voting method described for the initial round. For this evaluation, the panel members were provided with a summary of the opinions issued anonymously in the first round in addition to any other information that the scientific committee deemed appropriate to make available to the panelists to achieve consensus.

After analysis of the responses as described for round 1, the statements not meeting expert agreement were retained for discussion.

#### 5.2.5. Concluding Round

The concluding round comprised a teleconference meeting among the scientific committee experts to assess those items that did not reach consensus in round 2. The scientific committee members discussed the non-consensus items until an agreement was reached to either retain or eliminate the item from the final consensus guidelines.

### 5.3. Statistical Analysis

A descriptive statistical analysis of the data obtained from the expert panel’s assessment of the Delphi questionnaire items in rounds 1 and 2 was conducted. The distribution of frequencies of panel responses on the 9-point scale was calculated to establish the level of consensus for each questionnaire item.

A descriptive statistical analysis of the characteristics of the Delphi expert panel was also performed, including calculation of central tendency and dispersion (mean ± standard deviation, median, and interquartile range) for quantitative variables and frequencies and valid percentages for qualitative variables.

The statistical analysis was performed using the Statistical Package for the Social Sciences (SPSS) version 18.0 (SPSS Inc., Chicago, IL, USA).

## Figures and Tables

**Table 1:** Characteristics of the Delphi expert panel.

Characteristics	(N = 47)
**Age**, median (range), years	49.0 (32.0–66.0)
**Gender**, female, n (%)	26 (55.3)
**Professional experience**, median (range), years	22.0 (4.0–41.0)
**Specialty**, n (%)	
Physical Medicine and Rehabilitation	47 (100)
**Type of hospital**, n (%)	
Public hospital	42 (89.4)
Private hospital	3 (6.4)
Other	2 (4.2)
**Number of neurologically affected patients/week, median (range)**	20.0 (6.0–75.0)
**Resources related to the management of HSP patients**, n (%)	
Ultrasound scanner	47 (100)
Spasticity consultation	36 (76.6)
Auxiliary nurse service	34 (72.3)

**Table 2:** Risk factors for the development of post-stroke hemiplegic shoulder pain.

Risk Factor	Agreement [Scores 7–9] (%)
Impairment of voluntary motor control	93.6
Spasticity in flexor, adductor, and shoulder internal rotator muscles	93.5
Proprioceptive dysfunction	91.5
Range of motion (ROM) restriction to flexion, abduction, and external rotation	89.4
Tactile extinction	83.0
Prior anomalies	80.9

**Table 3:** Factors that should be considered in selecting the therapeutic approach to HSP secondary to stroke.

Risk Factor	Agreement [Scores 7–9] (%)
Presence or absence of pain/pain intensity	100
Presence or absence of spasticity	91.5
Cause of pain (neurological vs. mechanical factors)	91.5
Comorbidity	87.2
Degree of motor impairment	83.0
Time since stroke	74.5

**Table 4:** Recommended dosage for the muscles most frequently injected with abobotulinumtoxinA.

Muscle	Agreement [Scores 7–9] (%)	Recommended Dose per Session to Infiltrate Each Muscle [Minimum–Maximum] (U)	Agreement [Scores 7–9] (%)	Points of Muscle Administration	Agreement [Scores 7–9] (%)
Pectoralis major	100	150–300	95.7	At least 2 infiltration points	74.5
Subscapularis	95.7	150–300	97.8	A single infiltration point	95.7
Latissimus dorsi	68.1	150–300	93.5	At least 2 infiltration points	74.5
Teres major	68.1	Not approved in Spain	N/A	A single infiltration point	91.5

Abbreviations: U = units.

**Table 5:** Recommendations for abobotulinumtoxinA administration inf post-stroke HSP.

Recommendation	Agreement (A) [Scores 7–9] Disagreement (D) [Scores 1–3] (%)
Administration of ≤1 mL per injection site	67.4
Dilute 500 U aboBoNT-A in 2 mL	82.6
Shoulder muscles dose range of 600–1200 U	67.4
Shoulder muscles dose range of 1200–1500 U	69.6 (D)
In certain cases, it may be advisable to administer higher doses per muscle than those indicated in the SmPC	80.4
Recommended dose per muscle per session: 150–300 U	
Pectoralis major	95.7
Subscapularis	97.8
Latissimus dorsi	93.5
Optimal time between injections: 12–16 weeks	80.0

**Abbreviations:** aboBoNT-A = abobotulinumtoxinA; mL = milliliters; SmPC = summary of product characteristics; U = units.

## Data Availability

Qualified researchers may request access to patient-level study data that underlie the results reported in this publication. Additional relevant study documents, including the clinical study report, study protocol with any amendments, annotated case report form, statistical analysis plan and dataset specifications may also be made available. Patient-level data will be anonymized, and study documents will be redacted to protect the privacy of study participants. Where applicable, data from eligible studies are available 6 months after the studied medicine and indication have been approved in the US and EU or after the primary manuscript describing the results has been accepted for publication, whichever is later. Further details on Ipsen’s sharing criteria, eligible studies and process for sharing are available here (https://vivli.org/members/ourmembers/). Any requests should be submitted to www.vivli.org for assessment by an independent scientific review board.

## Supplementary Materials

The following supporting information can be downloaded at https://www.mdpi.com/article/10.3390/toxins17010040/s1, Figure S1: Geographical distribution throughout Spain of Delphi panelists in rounds 1 and 2; Table S1: Results of the two-step Delphi process for the items regarding the diagnosis of post-stroke focal spasticity; Table S2: Results of the two-step Delphi process for the items regarding to the treatment of hemiplegic shoulder pain (HSP); Table S3: Results of the two-step Delphi process for the items regarding to the follow-up of the post-stroke hemiplegic shoulder pain (HSP) patient treated with botulinum toxin A (BoNT-A).
