# Supplementary material for: Delphi Consensus on the Management of Spanish Patients with Post-Stroke Hemiplegic Shoulder Pain Treated with Botulinum Toxin A: Result Study
Source: Toxins (Basel). 2025 Jan 16;17(1):40. doi: 10.3390/toxins17010040 (PMC11769552; doi:10.3390/toxins17010040)
Supplement: Supplementary file 1 [file toxins-17-00040-s001.zip › toxins-3291883-supplementary.pdf]

Region	n (%)
Andalucía	8 (17.0)
Galicia	7 (14.9)
Canarias	6 (12.8)
Madrid	6 (12.8)
Cataluña	5 (10.6)
Comunidad Valenciana	4 (8.5)
Castilla y León	3 (6.4)
Baleares	2 (4.3)
País Vasco	2 (4.3)
Asturias	1 (2.1)
Cantabria	1 (2.1)
Murcia	1 (2.1)
Navarra	1 (2.1)
**Total**	**47**

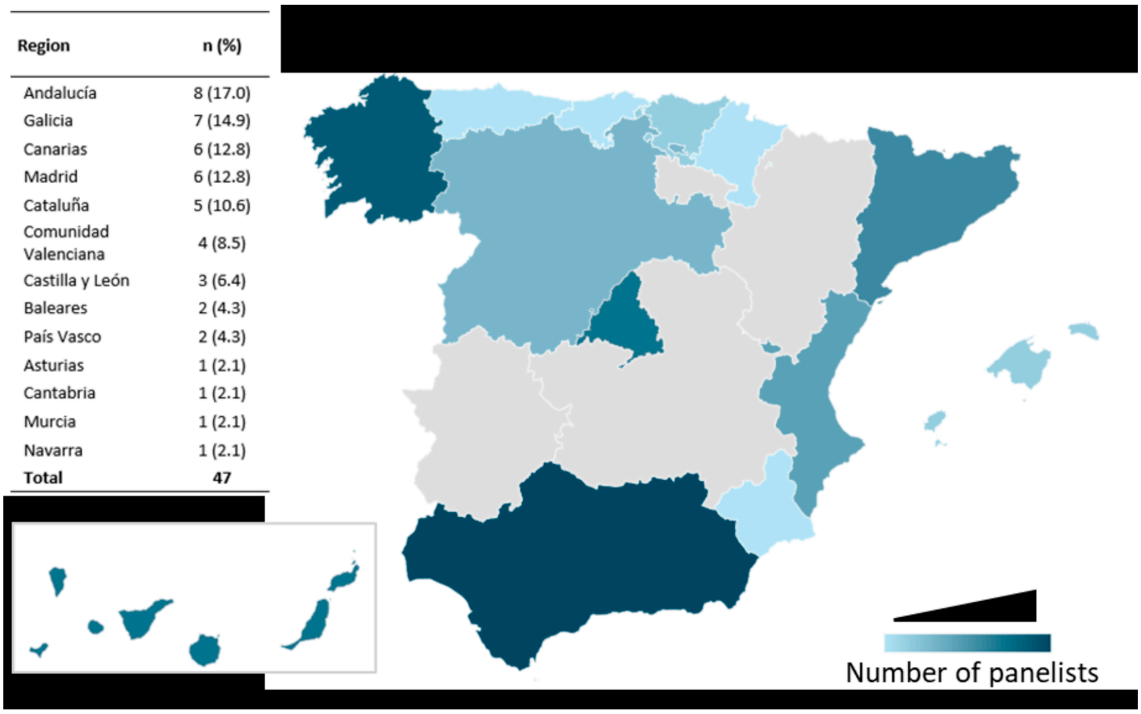

Figure S1: Geographical distribution throughout Spain of Delphi panelists in rounds 1 and 2

Table S1: Results of the two-step Delphi process for the items regarding the diagnosis of post-stroke focal spasticity.

**Items**	**Median (range)**	**Agreement (A) [scores 7-9] Disagreement (D) [scores 1-3] (%)**	**Delphi process outcome**
1. After stroke, close monitoring of patients is recommended in order to identify spasticity as early as possible.	9 (8-9)	95.7%	Agreed in round 1
Risk factors for the development of post-stroke hemiplegic shoulder pain (HSP) include:			
2. Age	4 (2-7)	33.3	Not agreed in round 2
3. Proprioceptive dysfunction	8 (7-9)	91.5	Agreed in round 1
4. Tactile extinction	8 (7-9)	83	Agreed in round 1
5. Impairment of voluntary motor control	9 (8-9)	93.6	Agreed in round 1
6. Spasticity in flexor, adductor, shoulder internal rotator muscles	9 (8-9)	93.5	Agreed in round 2 after being reformulated

7. Range of motion (ROM) restriction to flexion, abduction and external rotation	9 (8-9)	89.4	Agreed in round 1
8. Prior anomalies	8 (7-9)	80.9	Agreed in round 1
9. There are no well-established criteria for the diagnosis of post-stroke HSP.	7 (7-8)	76.6	Agreed in round 1
The diagnosis of HSP should include:			
10. Review of medical records	9 (9-9)	100	Agreed in round 1
11. Physical examination	9 (9-9)	100	Agreed in round 1
12. Imaging tests: ultrasound scan	8 (7-9)	76.6	Agreed in round 1
13. Imaging tests: X-ray	7 (5-9)	68.1	Agreed in round 1
14. Imaging tests: Magnetic Resonance Imaging (MRI)	3 (2-5)	51.1 (D)	Not agreed in round 1 Rejected
15. Electrodiagnostics	3 (2-6)	51.1 (D)	Not agreed in round 1 Rejected
16. Diagnostic Nerve Blocks (in selected cases)	7 (5-8)	65.2	Not agreed in round 2 after being reformulated Agreed after final evaluation by the scientific committee
Considering that the presence of HSP is generally of mixed origin, the most frequent aetiology is:			
17. Flaccid	3 (1-4)	73.9 (D)	Disagreed in round 2 after being reformulated
18. Central	3 (2-5)	54.3 (D)	Not agreed in round 2 after being reformulated Rejected
19. Spastic	7 (6-8)	71.7	Agreed in round 2 after being reformulated
20. Mechanical	6 (3-7)	47.8	Not agreed in round 2 after being

			reformulated Rejected
Initial assessment of the post-stroke HSP patient should include the following aspects:			
21. Spasticity	9 (9-9)	100	Agreed in round 1
22. Pain	9 (9-9)	100	Agreed in round 1
23. Muscular balance	9 (9-9)	97.9	Agreed in round 1
24. Motor control	9 (8-9)	97.9	Agreed in round 1
25. Range of motion (ROM) (extension, abduction and / or external rotation)	9 (9-9)	97.9	Agreed in round 1
26. Functionality (active/passive functions)	9 (9-9)	95.7	Agreed in round 1
27. Quality of life	9 (8-9)	89.4	Agreed in round 1
With regard to HSP, the following aspects should be considered:			
28. A differential diagnosis of pain should be made by assessing possible neurological and/or mechanical causes.	9 (9-9)	100	Agreed in round 1
29. It is important to treat pain as early as possible to avoid complications associated with post-stroke HSP.	9 (9-9)	100	Agreed in round 1
30. In most cases the origin of HSP is multifactorial.	9 (9-9)	97.9	Agreed in round 1
Following a stroke, the initial assessment of the HSP patient should include the use of the following scales/questionnaires:			
Pain assessment:			
31. Visual Analogue Scale (VAS)	9 (8-9)	93.6	Agreed in round 1
32. McGill Pain Questionnaire	7 (5-7)	53.2	Not agreed in round 1 Rejected
33. Spasticity-Associated Arm Pain Scale (SAAPS)	7 (5-8)	61.7	Not agreed in round 1 Rejected
Spasticity assessment:			
34. Modified Ashworth Scale (MAS)	9 (8-9)	100	Agreed in round 1

35. Ashworth Scale shoulder sum score (AS-SSS)	8 (6-9)	70.2	Agreed in round 1
36. Tardieu Scale	7 (6-8)	63.8	Not agreed in round 1 Rejected
37. Modified Tardieu Scale	7 (5-8)	55.3	Not agreed in round 1 Rejected
Motor function assessment:			
38. Fugl-Meyer Assessment (FMA)	7 (6-9)	68.1	Agreed in round 1
39. Brunnstrom Stages	7 (6-8)	74.5	Agreed in round 1
Quality of life assessment:			
40. EQ-5D	7 (6-8)	70.2	Agreed in round 1
41. SF-36	6 (5-7)	46.8	Not agreed in round 1 Rejected
42. SQoL-6D	7 (5-7)	59.6	Not agreed in round 1 Rejected

Table S2: Results of the two-step Delphi process for the items regarding to the treatment of hemiplegic shoulder pain (HSP).

Items	Median (range)	Agreement (A) [scores 7-9] Disagreement (D) [scores 1-3] (%)	Delphi process outcome
Factors that should be considered in selection of the therapeutic approach to HSP secondary to stroke include (but are not limited to):			
1. Age	1.5 (1-3)	80.4	Disagreed in round 2
2. Presence or absence of pain / pain intensity	9 (9-9)	100	Agreed in round 1
3. Presence or absence of spasticity	9 (9-9)	91.5	Agreed in round 1
4. The cause of pain (neurological vs. mechanical factors)	9 (8-9)	91.5	Agreed in round 1
5. Degree of motor impairment	8 (7-9)	83.0	Agreed in round 1
6. Time since stroke	7 (6-9)	74.5	Agreed in round 1
7. Comorbidity	8 (7-9)	87.2	Agreed in round 1
After identification of post-stroke HSP spasticity, the following invasive therapeutic procedures should be considered:			
8. Dry puncture of trigger points	5 (1-6)	42.6 (D)	Not agreed in round 1 Rejected
9. Infiltration of anti-inflammatory agents	5 (2-7)	41.3	Not agreed in round 2 Rejected
10. Botulinum toxin injections	9 (9-9)	100	Agreed in round 1
11. Nerve Block	8 (7-9)	89.4	Agreed in round 1
12. Hydrodilatation	5 (2-7)	34.0 (D)	Not agreed in round 1 Rejected
13. Radiofrequency	8 (7-9)	78.7	Agreed in round 1
14. Shock waves	3 (2-7)	56.5 (D)	Not agreed in round 2 Rejected

15. Surgical procedure	1 (1-2)	95.7	Disagreed in round 2
16. Other procedures	8 (7-9)	100	Agreed in round 1
Following identification of post-stroke HSP spasticity, the following non-invasive therapies/procedures should be considered:			
17. NSAID	5 (2-7)	38.3 (D)	Not agreed in round 1 Rejected
18. Opioids	3 (2-5)	63.0 (D)	Not agreed in round 2 Rejected
19. Electrotherapy	2.5 (2-6)	58.7 (D)	Not agreed in round 2 Rejected
20. Functional neurostimulation	3 (2-5)	56.5 (D)	Not agreed in round 2 after being reformulated Rejected
21. Ultrasound therapy	5 (1-7)	46.8 (D)	Not agreed in round 1 Rejected
22. Orthosis	4 (2-7)	50.0 (D)	Not agreed in round 2 Rejected
23. Acupuncture	2 (1-5)	57.4 (D)	Not agreed in round 1 Rejected
24. Non-invasive brain stimulation	5 (2-5)	40.4 (D)	Not agreed in round 1 Rejected
25. Physical therapy	9 (7-9)	89.4	Agreed in round 1
26. Other procedures	8 (7-9)	100	Agreed in round 1
After stroke, treatment of HSP spasticity with botulinum toxin should be initiated:			
27. Once spasticity has been identified and the spastic pattern has already been established	5 (2-7)	41.3	Not agreed in round 2 after being reformulated Rejected
As soon as post-stroke spasticity (even early, within the first 3 months after the event):			

28. Is bothersome for the patient	9 (7-9)	93.6	Agreed in round 1
29. Interferes with the patient's activities of daily living	9 (9-9)	100	Agreed in round 1
30. Causes pain	9 (9-9)	97.9	Agreed in round 1
31. Affects the patient's functional ability	9 (9-9)	93.6	Agreed in round 1
32. Results in a reduction of the patient's active and/or passive mobility	9 (8-9)	93.6	Agreed in round 1
The muscles most frequently infiltrated with botulinum toxin are:			
33. Pectoralis major	9 (9-9)	100	Agreed in round 1
35. Subscapularis	9 (9-9)	95.7	Agreed in round 1
36. Teres major	7 (6-8)	68.1	Agreed in round 1
37. Latissimus dorsi	7 (6-8)	68.1	Agreed in round 1
38. Other	6.5 (6-7)	50.0	Not agreed in round 1 Rejected
The following factors should be considered for selection of the muscle to be infiltrated with botulinum toxin:			
39. Spasticity degree	9 (9-9)	95.7	Agreed in round 1
40. Range of movement	9 (7-9)	89.4	Agreed in round 1
41. Pain	9 (9-9)	95.7	Agreed in round 1
42. Echogenicity degree	3 (2-6)	56.5 (D)	Not agreed in round 2 Rejected
43. Difficulty accessing the muscle	2 (1-3)	76.1	Disagreed in round 2
The muscles that are most difficult to access for infiltration with botulinum toxin are:			
44. Pectoralis major	1 (1-1)	89.4	Disagreed in round 1
46. Subscapularis	7 (3-8)	55.6	Not agreed in round 2 Rejected
47. Teres major	2 (1-6)	68.1	Disagreed in round 1
48. Latissimus dorsi	2 (1-6)	68.1	Disagreed in round 1

49. In most cases the route of administration should be intramuscular.	9 (9-9)	97.9	Agreed in round 1
Apart from intramuscular administration, the following routes of administration may be considered for infiltration with botulinum toxin:			
50. Intrabursal injection	2 (1-6)	61.7 (D)	Not agreed in round 1 Rejected
51. Intra-articular injection	5 (1-7)	38.3 (D)	Not agreed in round 1 Rejected
Regarding the points of muscle administration of botulinum toxin, the following could be considered for each muscle:			
Pectoralis major:			
52. a single infiltration point	5 (2-8)	43.5 (D)	Not agreed in round 2 Rejected
53. at least 2 infiltration points	8 (6-9)	74.5	Agreed in round 1
Triceps:			
54. a single infiltration point	5 (1-7)	44.7 (D)	Not agreed in round 1 Rejected
55. at least 2 infiltration points	9 (7-9)	85.1	Agreed in round 1
Subscapularis:			
56. a single infiltration point	9 (8-9)	95.7	Agreed in round 1
57. at least 2 infiltration points	4 (1-7)	44.7 (D)	Not agreed in round 1 Rejected
Teres major:			
58. a single infiltration point	9 (8-9)	91.5	Agreed in round 1
59. at least 2 infiltration points	3 (1-7)	51.1 (D)	Not agreed in round 1 Rejected
Latissimus dorsi:			
60. a single infiltration point	6 (1-8)	46.8	Not agreed in round 1 Rejected
61. at least 2 infiltration points	8 (6-9)	74.5	Agreed in round 1
62. Guidance techniques for infiltration should be used to improve the accuracy of botulinum toxin delivery.	9 (9-9)	100	Agreed in round 1

The most accurate guidance techniques for botulinum toxin infiltration are:			
63. Electromyography	2 (1-3)	82.2	Disagreed in round 2
64. Ultrasound scan	9 (9-9)	100	Agreed in round 1
65. Electrical stimulation	2 (1-3)	78.3	Disagreed in round 2
66. Anatomical guidance	4 (2-6)	40.4 (D)	Not agreed in round 1 Rejected
67. Muscle echogenicity should be considered to guide infiltration with botulinum toxin.	7 (7-9)	76.1	Agreed in round 1
Muscle echogenicity should be assessed using the following tools:			
68. Heckmatt Scale	8 (7-9)	83.0	Agreed in round 1
69. Heckmatt modified scale	8 (7-9)	83.0	Agreed in round 1
With regard to the intensity of echogenicity, infiltration with botulinum toxin should be performed in areas of:			
70. Lower echogenicity	9 (7-9)	85.1	Agreed in round 1
71. Higher echogenicity	2 (1-4)	71.7	Disagreed in round 2
72. Indistinctly	4 (2-7)	47.8 (D)	Not agreed in round 2 Rejected
The dose of botulinum toxin for management of the post-stroke HSP patient should be selected on the basis of:			
73. Spasticity degree	9 (7-9)	95.7	Agreed in round 1
Time since stroke:			
74. should be different during the acute and sub-acute phase	5 (1-7)	40.4	Not agreed in round 1 Rejected
75. should be different during the sub-acute and chronic phase	5 (1-7)	41.3 (D)	Not agreed in round 1 Rejected
76. Number, type and location of involved muscles	9 (8-9)	95.7	Agreed in round 1
77. Echogenicity degree	2 (1-3)	78.3	Disagreed in round 2
78. Physician expertise	2.5 (1-5)	67.4	Disagreed in round 2

79. Whether the patient has been previously treated with toxin or not	8 (7-9)	76.6	Agreed in round 1
The following factors/aspects should be considered in selection of the type of botulinum toxin for management of the post-stroke HSP patient:			
80. Efficacy or control of symptoms	9 (8-9)	87.0	Agreed in round 1
Therapeutic response curve:			
81. onset of effect	9 (7-9)	76.1	Agreed in round 1
82. duration of effect	9 (8-9)	91.3	Agreed in round 1
83. Safety (adverse events)	9 (8-9)	91.3	Agreed in round 1
84. Habit/ease of dose	7.5 (6-9)	73.9	Agreed in round 1
85. Maximum permitted dose	9 (7-9)	91.3	Agreed in round 1
86. Possibility to inject a larger number of muscles	9 (8-9)	87.0	Agreed in round 1
87. Pharmacy availability	8 (5-9)	71.7	Agreed in round 1
88. Others	8 (8-8)	100	Agreed in round 1
89. There is an unmet need for the optimal use of abobotulinumtoxinA, with a different dosage type than the other botulinum toxins available.	5 (1-7)	41.3	Not agreed in round 1 Rejected
In the context of abobotulinumtoxinA administration for post-stroke HSP management:			
90. In general, no more than 1 ml should be administered at a single injection site.	7 (6-9)	67.4	Agreed in round 1
In general, the maximum total dose range to be distributed to the shoulder muscles in each session should be:			
91. 300-600 U	4 (2-7)	47.8 (D)	Not agreed in round 2 Rejected
92. 600-1200 U	7 (4-8)	67.4	Agreed in round 2
93. 1200-1500 U	2 (1-4)	69.6	Disagreed in round 2
94. The maximum dose per muscle should not exceed 300 U	7.5 (6-9)	73.9	Agreed in round 1

The recommended dose of abobotulinumtoxinA per session for infiltrating each muscle should be as follows:			
95. Pectoralis major: 150-300 U	9 (8-9)	95.7	Agreed in round 1
96. Triceps: 150-300 U	9 (7-9)	84.8	Agreed in round 1
97. Subscapularis: 150-300 U	9 (8-9)	97.8	Agreed in round 1
98. Latissimus dorsi: 150-300 U	9 (8-9)	93.5	Agreed in round 1
The recommended dilution per 500 U of abobotulinumtoxinA is:			
99. 1 ml	6 (1-8)	43.5	Not agreed in round 1 Rejected
100. 2 ml	9 (7-9)	82.6	Agreed in round 1
101. 2.5 ml	7 (5-8)	60.9	Not agreed in round 1 Rejected
102. 2.5-5 ml	6 (2-8)	43.5	Not agreed in round 1 Rejected
103. 5-10 ml	2.5 (1-6)	58.7 (D)	Not agreed in round 1 Rejected
104. In some cases, it may be advisable to administer higher doses per muscle than those indicated in the Summary of Product Characteristics.	8 (7-9)	80.4	Agreed in round 1
The optimal time between botulinum toxin injections should be:			
105. 12 weeks	7 (5-8)	58.7	Not agreed in round 1 Rejected
106. 12-16 weeks	8 (7-8)	80.0	Agreed in round 2
107. 20-24 weeks	6 (3-7)	37.0	Not agreed in round 1 Rejected
108. From 12 weeks onwards, when the patient reports that the effect of the botulinum toxin has disappeared/decreased, regardless of the interval established according to local clinical practice.	8 (6-9)	73.9	Agreed in round 2 after being reformulated

To achieve optimal benefit, botulinum toxin infiltration must be accompanied by:			
109. Orthosis	3 (2-4)	73.9	Disagreed in round 2
110. Casts	3 (1-6)	54.3 (D)	Not agreed in round 1 Rejected
111. Home-based exercises	9 (8-9)	91.3	Agreed in round 1
112. Physiotherapy	8 (7-9)	80.4	Agreed in round 1

Table S3: Results of the two-step Delphi process for the items regarding to the follow-up of the post-stroke hemiplegic shoulder pain (HSP) patient treated with botulinum toxin A (BoNT-A).

Items	Median (range)	Agreement (A) [scores 7-9] Disagreement (D) [scores 1-3] (%)	Delphi process outcome
Follow-up of the HSP patient treated with botulinum toxin should be multidisciplinary, involving:			
1. Neurology	5 (1-7)	43.5 (D)	Not agreed in round 1 Rejected
2. Physical medicine and rehabilitation	9 (9-9)	100	Agreed in round 1
3. Others	8 (8-8)	80.0	Agreed in round 1
4. After the startt of botulinum toxin treatment for the management of HSP, it is important to follow-up on the patient to confirm that the established treatment goals have been reached.	9 (9-9)	100	Agreed in round 1
Regarding the frequency of follow-up of the HSP patient after the first infiltration of botulinum toxin:			
5. First follow-up of the patient should take place 4-6 weeks after the first infiltration.	9 (8-9)	95.7	Agreed in round 1
6. The frequency should be determined by the duration of the botulinum toxin effect.	8 (6-9)	73.9	Agreed in round 1
7. The frequency should be determined by the patient's needs (rather than on the basis of pre-defined regimens).	8 (6-9)	73.9	Agreed in round 1
8. During the first year of botulinum toxin treatment, follow-up is recommended every 12-16 weeks.	8 (7-9)	84.8	Agreed in round 1
9. After the first year of botulinum toxin treatment, follow-up should be individualized for each patient considering the response times and duration of effect.	9 (8-9)	97.8	Agreed in round 1

10. The goals of botulinum toxin treatment in HSP may change during patient follow-up.	9 (9-9)	100	Agreed in round 1
Treatment goals may change during the patient follow-up depending on:			
11. the stage of the patient (sub-acute or chronic)	8 (7-9)	84.8	Agreed in round 1
12. evolution of the spasticity pattern	9 (8-9)	97.8	Agreed in round 1
13. evolution of the patient's range of motion	8 (7-9)	87.0	Agreed in round 1
14. evolution of the patient's functionality	9 (8-9)	91.3	Agreed in round 1
15. the patient's ability to perform activities of daily living	9 (8-9)	91.3	Agreed in round 1
16. pain evolution	9 (8-9)	97.8	Agreed in round 1
17. evolution of muscular echogenicity	6 (4-7)	43.5	Not agreed in round 1 Rejected
18. patient quality of life	9 (7-9)	91.3	Agreed in round 1
19. Treatment goals should be reassessed at each follow-up visit.	9 (9-9)	100	Agreed in round 1
20. Use of the Goal Attainment Scaling (GAS) is recommended for reassessment of targets during patient follow-up.	9 (7-9)	84.8	Agreed in round 1
The treatment plan should be re-evaluated at each follow-up visit with respect to:			
21. treatment goals	9 (9-9)	97.8	Agreed in round 1
22. treatment response	9 (9-9)	97.8	Agreed in round 1
23. treatment-associated adverse events	9 (9-9)	97.8	Agreed in round 1
24. treatment goals, treatment response, and treatment-associated adverse events	9 (9-9)	97.8	Agreed in round 1
25. patient autonomy	7 (7-9)	78.3	Agreed in round 1
26. the response to diagnostic nerve blockades (if any)	8 (7-9)	87.0	Agreed in round 1
Patient assessment at each follow-up visit should include evaluation of the following aspects:			

27. evolution of the spasticity degree and spasticity pattern	9 (9-9)	97.8	Agreed in round 1
28. pain (presence and intensity)	9 (9-9)	100	Agreed in round 1
29. motor function	9 (8-9)	89.1	Agreed in round 1
30. range of motion (ROM) (flexion, external abduction and/or rotation)	9 (8-9)	93.5	Agreed in round 1
31. functionality (active/passive functions)	9 (8-9)	95.7	Agreed in round 1
32. quality of life	9 (7-9)	89.1	Agreed in round 1
33. evolution and GAS goals established in the previous infiltration.	9 (7-9)	89.1	Agreed in round 1
34. It is advisable to use validated scales to assess the evolution of the patient treated with botulinum toxin.	9 (8-9)	93.5	Agreed in round 1
The use of the following scales is recommended to assess the patient evolution:			
Pain assessment			
35. Visual Analogue Scale (VAS)	9 (8-9)	97.8	Agreed in round 1
36. McGill Pain Questionnaire	7 (5-7)	56.5	Not agreed in round 1 Rejected
37. Spasticity-Associated Arm Pain Scale (SAAPS)	7 (5-8)	65.2	Not agreed in round 1 Rejected
Spasticity assessment			
38. Modified Ashworth Scale (MAS)	9 (8-9)	100	Agreed in round 1
39. Ashworth Scale shoulder sum score (AS-SSS)	7 (7-8)	76.1	Agreed in round 1
40. Tardieu Scale	7 (6-8)	65.2	Not agreed in round 1 Rejected
41. Modified Tardieu Scale	7 (6-8)	71.7	Agreed in round 1
Motor function assessment			
42. Fugl-Meyer Motor (FM) Scale	7 (6-9)	65.2	Not agreed in round 1 Rejected
43. Brunnstrom Scale	7 (6-8)	71.7	Agreed in round 1
Quality of life assessment			
44. EQ-5D	7 (6-8)	73.9	Agreed in round 1

45. SF-36	6.5 (5-7)	50.0	Not agreed in round 1 Rejected
46. SQoL-6D	7 (5-7)	58.7	Not agreed in round 1 Rejected
47. It is advisable to use Patient Reported Outcomes (PROs) (e.g., symptoms diary) to assess the evolution of the patient treated with botulinum toxin.	7 (7-8)	78.3	Agreed in round 1
48. During follow-up of the HSP patient, it is advisable to assess caregiver burden (if the patient has a caregiver).	8 (7-9)	93.5	Agreed in round 1
49. Before administration of the next botulinum toxin infiltration, the patient should be asked about the incidence of possible adverse events after administration of the last dose.	9 (9-9)	100	Agreed in round 1
In a patient experiencing adverse events with botulinum toxin administration, the following actions should be taken:			
50. to reduce the dose	7 (5-9)	58.7	Not agreed in round 1 Rejected
51. to assess possible drug interactions (antibiotics, muscle relaxants, etc.)	9 (8-9)	93.5	Agreed in round 1
52. to continue the treatment if the reaction is mild and transient	7 (7-8)	76.1	Agreed in round 1
53. to withdraw the treatment	5 (2-7)	37.0 (D)	Not agreed in round 1 Rejected
54. to change the botulinum toxin formulation	6 (3-7)	45.7	Not agreed in round 1 Rejected
In a patient with a lack of response to botulinum toxin treatment, the following actions should be taken:			
55. to increase the dose	8 (7-9)	89.1	Agreed in round 1
56. to increase the number of treated muscles	8 (7-9)	84.8	Agreed in round 1
57. to optimize the muscle location to infiltrate	9 (8-9)	95.7	Agreed in round 1

58. to increase the dose and the number of treated muscles	8 (7-9)	87.0	Agreed in round 1
59. to maintain the same dose	2 (1-5)	65.2 (D)	Not agreed in round 1 Rejected
60. to change the dilution	6 (3.7)	45.7	Not agreed in round 1 Rejected
61. to withdraw the treatment	2 (1-5)	63.0 (D)	Not agreed in round 1 Rejected
62. to change the botulinum toxin formulation	5.5 (2-7)	39.1	Not agreed in round 1 Rejected
63. to assess pharmacological therapeutic alternative	7 (6-8)	63.0	Not agreed in round 1 Rejected
64. to assess non-pharmacological therapeutic alternatives	7 (6-8)	69.6	Agreed in round 1
65. others	9 (9-9)	100	Agreed in round 1
The main reasons for termination of botulinum toxin treatment include:			
66. lack of response to treatment	9 (7-9)	91.3	Agreed in round 1
67. spasticity progression	6 (2-7)	47.8	Not agreed in round 1 Rejected
68. failure to achieve the established goals	8 (7-9)	78.3	Agreed in round 1
69. treatment-associated adverse events	8 (6-9)	73.9	Agreed in round 1
70. others	9 (9-9)	100	Agreed in round 1
